# Structural Insights into the Effector – Immunity System Tae4/Tai4 from *Salmonella typhimurium*

**DOI:** 10.1371/journal.pone.0067362

**Published:** 2013-06-27

**Authors:** Juliane Benz, Jochen Reinstein, Anton Meinhart

**Affiliations:** Department of Biomolecular Mechanisms, Max Planck Institute for Medical Research, Heidelberg, Germany; Institut Pasteur Paris, France

## Abstract

Type-6-secretion systems of Gram-negative bacteria are widely distributed needle-like multi-protein complexes that are involved in microbial defense mechanisms. During bacterial competition these injection needles dispense effector proteins into the periplasm of competing bacteria where they induce degradation of the peptidoglycan scaffold and lead to cell lysis. Donor cells co-produce immunity proteins and shuttle them into their own periplasm to prevent accidental toxication by siblings. Recently, a plethora of previously unidentified hydrolases have been suggested to be peptidoglycan degrading amidases. These hydrolases are part of effector/immunity pairs that have been associated with bacterial warfare by type-6-secretion systems. The *Tae4* and *Tai4* operon encoded by *Salmonella typhimurium* is one of these newly discovered effector/immunity pairs. The Tae4 effector proteins induce cell lysis by cleaving the γ-D-glutamyl-L-*meso*-diaminopimelic acid amide bond of acceptor stem muropeptides of the Gram-negative peptidoglycan. Although homologues of the Tae4/Tai4 system have been identified in various different pathogens, little is known about the functional mechanism of effector protein activity and their inhibition by the cognate immunity proteins. Here, we present the high-resolution crystal structure of the effector Tae4 of *S. typhimurium* in complex with its immunity protein Tai4. We show that Tae4 contains a classical NlpC/P60-peptidase core which is common to other effector proteins of the type-6-secretion system. However, Tae4 has unique structural features that are exclusively conserved within the family of Tae4 effectors and which are important for the substrate specificity. Most importantly, we show that although the overall structure of Tai4 is different to previously described immunity proteins, the essential mode of enzyme inhibition is conserved. Additionally, we provide evidence that inhibition in the Tae4/Tai4 heterotetramer relies on a central Tai4 dimer in order to acquire functionality.

## Introduction

Pathogenic Gram-negative bacteria produce a multitude of toxic proteins that they either secrete into their environment or directly inject into target cells during their struggle for biological niches [1], [2]. Recently, an entire family of toxic effector proteins has been identified which are injected by the type-6-secretion system (T6SS) of various Gram-negative bacteria into the periplasmic space of competing cells [3], [4]. The T6SS injection apparatus by which pathogenic bacteria deliver these effector proteins into competing cells is a contractile needle-like injection system [5] composed of 13 core proteins [6]. It spans both bacterial membranes of Gram-negative bacteria and is structurally homologous to bacteriophage tails [5], [7], [8], [9], [10].

The majority of these novel toxic effector molecules degrade the peptidoglycan layer and are normally contained in the cytoplasm of their producer before injection. However, the producer’s own peptidoglycan is not inherently resistant to the toxic activity of these effector proteins and bacteria therefore co-produce specific cognate immunity proteins. These immunity proteins are shuttled into their own periplasmic space and prevent self-intoxication by strayed effector molecules or those injected by siblings [3], [4]. Normally, effector and immunity proteins are co-encoded on a bicistronic operon [3], [4]. However, genes of immunity proteins alone without the effector genes have been identified as well, suggesting that bacteria use them to protect themselves against attacks from foreign species [4], [11].

Although T6SSs are widespread among Gram-negative bacteria [12] to date only a few effector proteins have been characterized. The best studied effector/immunity systems are the type-6-secretion effectors Tse1/Tsi1, Tse2/Tsi2, and the Tse3/Tsi3 systems from *Pseudomonas aeruginosa.* Tse1–3 are substrates of the haemolysin co-regulated protein secretion island I -encoded T6SS from *P. aeruginosa* and are injected into rival cells to provide an advantage during bacterial growth competition [3], [13].

Whereas the Tse1 effector was shown to cleave the γ-D-glutamyl-L-*meso*-diaminopimelic acid amide bond of cross-linked muropeptides, Tse3 was described to cleave the peptidoglycan backbone between the *N*-acetylmuramine and *N*-acetylglucosamine sugar moieties [3]. In contrast, the activity employed by Tse2 to kill recipient cells is still unknown.

**Figure 1:**
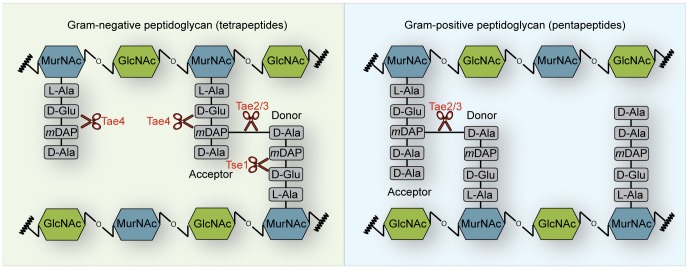
Schematic representation showing the peptidoglycan cleavage specificities of the type-6-secretion amidase (Tae) effector families. Cleavage specificity of the Tae1–4 effector proteins on Gram-negative tetrapeptide stems (left) or Gram-positive pentapeptide stems (right) is depicted based on previous reports [3], [4]. Tae2 and Tae3 effector proteins hydrolyze the amide bond between the *m*DAP-D-Ala cross-link in Gram-negative as well as Gram-positive peptidoglycan. In contrast, members of the Tae1 (including Tse1 from *P. aeruginosa*) and the Tae4 effector family degrade the peptidoglycan scaffold by cleaving the γ-D-glutamyl-*m*DAP bond. Whereas, Tae4 exclusively hydrolyzes the acceptor stem of cross-linked as well as non-cross-linked Gram-negative peptidoglycan, Tse1 specifically cleaves the cross-linked donor peptide stem. Furthermore, Gram-positive peptidoglycan is a poor substrate for Tae4 family members.

A plethora of previously unrecognized peptidoglycan hydrolases has been identified as potential effector proteins that are encoded together with their cognate immunity proteins from bicistronic operons in different bacteria [4]. All of these effector proteins belong to the family of NlpC/P60 papain-like cysteine peptidases and are amidases that cleave between the muropeptide moieties of peptidoglycan. These new amidases have been grouped into four families (type-6-secretion amidase effector 1–4) according to their cleavage specificity (Figure 1) [4].

Effector proteins belonging to the Tae2 or the Tae3 family specifically cleave the L-*meso*-diaminopimelic-acid-D-alanine cross-link of muropeptides within peptidoglycan from Gram-negative but also from Gram-positive bacteria (Figure 1). In contrast, Tae1 effectors including its founding member Tsi1 from *P. aeruginosa* exclusively cleave the γ-D-glutamyl-L-*meso*-diaminopimelic acid amide bond at the donor stem side of cross-linked peptidoglycan of Gram-negative bacteria (Figure 1). Tae4 cleaves the γ-D-glutamyl-L-*meso*-diaminopimelic acid amide bond as do Tae1 members; however, it specifically hydrolyzes the acceptor stem of the muropeptides (Figure 1).

To date, only the Tse1/Tsi1 effector/immunity pair from *P. aeruginosa* has yielded structural information [14], [15], [16]. We therefore determined the three-dimensional structure of the Tae4/Tai4 effector/immunity system from *Salmonella typhimurium* by X-ray crystallography. The Tae4 structure displays several distinct features when compared with its structural relative Tse1. These features are highly conserved within all known members of this family and thus most likely are responsible for the different substrate specificity of the two effector proteins. Moreover, the immunity protein Tai4 represents a new family of T6SS immunity proteins forming a heterotetramer with its cognate effector Tae4. Finally, we provide evidence that a similar effector/immunity protein assembly also exists in other bacteria and that the mode of Tai4 inhibition is conserved. Shortly before this manuscript was submitted, similar crystal structures of the Tae4/Tai4 protein complex from *S. typhimurium* and *Enterobacter cloacae* were reported [17].

## Results and Discussion

### Crystallization and Structure Determination

Crystals of the effector/immunity system Tae4/Tai4 from *S. typhimurium* readily grew to a size of 400×100×100 µm^3^ within 3 days in 2-ethoxyethanol containing conditions. Experimental phases were obtained from a Single-wavelength Anomalous Diffraction (SAD) experiment and an initial structure was built and refined to a resolution of 2.3 Å. Native protein crystals of identical crystal symmetry diffracted to a resolution of 1.8 Å at the Swiss Light Source (Villigen, CH), allowing the refinement of a structural model at near atomic resolution. Both models were finally refined to excellent stereochemistry, with 97% of the residues in preferred regions of the Ramachandran plot. Statistics of data collection, structure refinement and model quality can be found in Table 1.

**Table 1:** Data collection and refinement statistics.

Crystal	Tae4_Tai4 native	Tae4_Tai4 SeMet
**Wavelength (Å)**	0.99981	0.97933
**Spacegroup**	*P*6_1_22	*P*6_1_22
**Cell parameters**	*a = *63.65, *b* = 63.65, *c* = 365.65, *α* = 90, *β* = 90, γ = 120	*a = *63.38, *b* = 63.38, *c* = 364.99, *α* = 90, *β* = 90, γ = 120
**Resolution (highest shell) (Å)**	40.0–1.8 (1.9-1.8)	40.0–2.3 (2.4-2.3)
**# reflections, all**	761,089 (115,546)	1,535,040 (187,499)
**# reflections, unique**	42,126 (6,089)	36,560 (4,358)
**Completeness (%)**	99.1 (98.6)	99.9 (99.9)
**Redundancy**	18.1 (19.0)	42.0 (43.0)
**I/σ(I)**	18.7 (4.7)	24.0 (7.1)
**R_merge_**	10.2 (66.1)	15.4 (58.7)
**Resolution range in refinement**	37.9–1.8	37.8–2.3
**R_work_/R_free_**	0.183/0.203	0.198/0.231
**# atoms**		
Protein (A/B)	1262/866	1205/828
Water	202	106
Others	26 (2 citrate) 48 (8 2-ethoxyethanol)	13 (1 citrate) 72 (12 2-ethoxyethanol)
**Average B-factors**		
Protein (A/B)	29.685/28.956	28.383/29.481
Water	34.495	31.570
Others	50.513	51.859
**Rmsd from target values**		
Bond lengths (Å)	0.013	0.010
Bond angles (°)	1.475	1.120
**%age of residues in region of Ramachandran plot :**		
Preferred	97.45	97.15
Allowed	2.55	2.85
Outliers	0.0	0.0

Values in parentheses are those for the highest resolution shell.

### The Tae4-effector from *Salmonella typhimurium*

**Figure 2:**
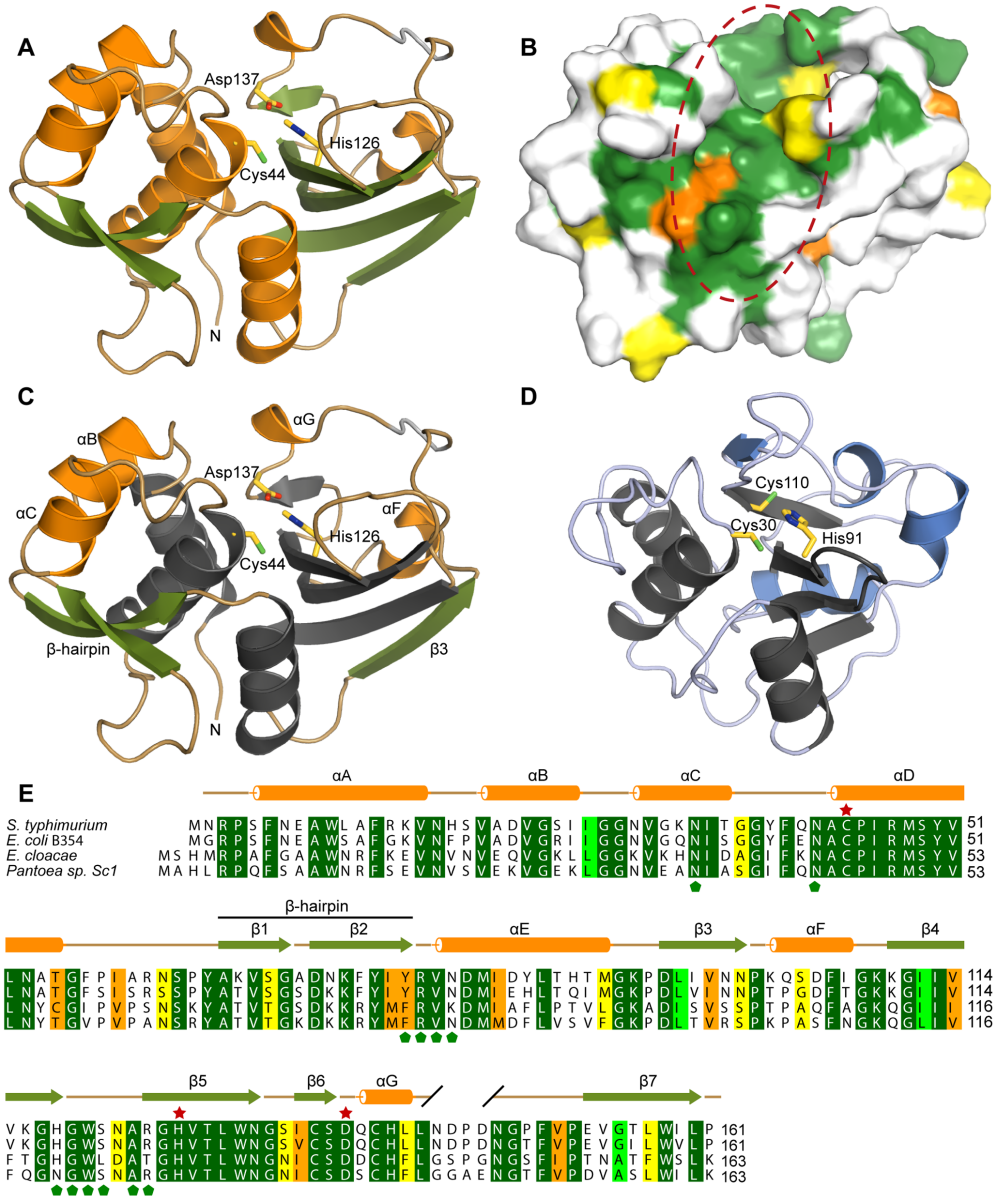
rystal structure and conservation of the Tae4 effector from *S. typhimurium*. (A) Ribbon representation of *S. typhimurium* Tae4. Tae4 adopts the classical papain-like fold of cysteine peptidases of the NlpC/P60 family. The core of the Tae4 effector protein is formed by a central anti-parallel β-sheet which is flanked by α-helices. Cys44, His126 and Asp137 are shown as a stick model. α-helices are shown in orange and β-strands are colored in green. The loop region between residue Asn143 and Asn147 which is not included in the model is indicated in gray. (B) Amino acid sequence conservation of Tae4 effector proteins mapped on the molecular surface colored according to (E). The crevice harboring the conserved active site residues is highlighted with a red dashed circle. (C) Crystal structure of the effector protein Tae4 with the conserved NlpC/P60 catalytic core in gray (same orientation as in (A)). The additional α-helices B, C, G and F are colored in orange. The Tae4-specific β-strands 1 and 2 forming the β-hairpin as well as the β-strand 3 are shown in green. Cys44, His126 and Asp137 are depicted as stick model. (D) Crystal structure of the effector protein Tse1 from *P. aeruginosa* [15] with the conserved NlpC/P60 catalytic core in gray. Tse1 specific elements are colored in blue. The Cys30, His91 and Cys110 residues similar to Tae4’s Cys44, His126 and Asp137 are depicted as stick model. (E) Amino acid sequence alignment of Tae4 effectors from *Salmonella typhimurium* (NP_459275.1), *Escherichia coli* B354 (ZP_06652154.1), *Enterobacter cloacae* (YP_006478116.1) and *Pantoea sp. Sc1* (ZP_09929551.1) going from dark green (identical residues) to light green, orange and yellow (with decreasing conservation). α-helices and β-strands are colored in orange and green according to (A). Residues of the Tae4 catalytic triad are marked with a red star. Green pentagons below the sequence alignment indicate surface exposed residues that interact with the Tai4 immunity proteins.

An almost complete Tae4 polypeptide chain comprising Asn2 through the C-terminal Pro161 could be unambiguously modeled into the electron density map and refined. Only the loop region between residue Asn143 and Asn147 had poor electron density and we therefore did not model it (Figure 2A).

The core of the Tae4 effector is formed by a central antiparallel β-sheet flanked by α-helices (Figure 2A). Thus, Tae4 adopts the classical papain-like fold of cysteine peptidases of the NlpC/P60 family [18]. A DALI search [19] revealed that aside from the evident homology to the homologous Tae4 from *E. cloacae* [17] (r.m.s.d of 1.5 Å and 54% sequence identity) the related peptidoglycan hydrolase effector Tse1 from *P. aeruginosa* [14], [15], [16], [20], [21] (r.m.s.d of 3.6 Å and 16% sequence identity) and the functionally unrelated NlpC/P60 domain of YkfC from *Bacillus cereus* [22] (r.m.s.d of 3.7 Å and 12% sequence identity) are the closest structural homologs of Tae4 from *S. typhimurium*.

As a true NlpC/P60 papain-like cysteine peptidase, Tae4 has a catalytically important cysteine residue (Cys44) in its active site. This Cys44 is in close proximity to His126, which most likely deprotonates and activates the cysteine thiol group (Figure 2A). Together, Cys44 and His126 establish a catalytic dyad, as described for Tse1 [14], [15], [16], [20], [21] and other NlpC/P60 hydrolases [18], [23]. The activated thiolate ion of Cys44 is expected to perform the nucleophilic attack on the amide bond of the peptidoglycan resulting in a tetrahedral oxyanion intermediate. This oxyanion is most likely stabilized by the backbone amide groups of Cys44 and Ile99 which form an oxyanion hole, as described for the catalytically related serine proteases [24], [25].

Somewhat further away from Cys44, Asp137 forms a hydrogen bond to the His126 imidazole ring (Figure 2A), reminiscent of the previously proposed catalytic triad of serine proteases [25]. In contrast, the effector protein Tse1 from *P. aeruginosa* and YkfC from *B. cereus* contain a cysteine or a histidine residue at the structurally equivalent position [14], [15], [16], [20], [21], [22] showing that NlpC/P60 papain-like cysteine peptidases do not require an aspartate residue at this particular position. Tae4 and Tse1 both cleave the γ-D-glutamyl-L-*meso*-diaminopimelic acid amide bond of the Gram-negative peptidoglycan scaffold [3], [4]. However, these two peptidoglycan hydrolases vary in accepting different stems of the cross-linked muropeptides. Whereas Tae4 exclusively recognizes and cleaves the γ-D-glutamyl-L-*meso*-diaminopimelic acid amide bond at the acceptor stem side of cross-linked Gram-negative peptidoglycan, Tse1 specifically cleaves at the donor peptide side [4] (Figure 1).

These differences in specificity may be explained by different muropeptide binding sites close to the active site. When comparing Tae4 and Tse1, differences in structural elements outside of the classical NlpC/P60 papain-like cysteine peptidase core domain became evident (Figure 2C and D). Firstly, Tae4 contains a significant number of long loop insertions when compared to Tsi1 (connecting αC with αD, β4 with β5 and αG with β7). These loop regions are adjacent to the Tae4 active site, forming the wall of a deep crevice with the active site located beneath (Figure 2A and B). In contrast, Tse1 exhibits a rather shallow muropeptide binding site [15]. Moreover, in Tae4 a β-hairpin (formed by β strand 1 and 2) is inserted between the helices αD and αE which closes over the active site. This β-hairpin is held in place by α-helices B and C, an additional structural feature that is different to Tse1 (Figure 2C and D).

Most importantly, an amino acid sequence alignment of related Tae4 proteins encoded from homologous bicistronic Tae4/Tai4 operons showed that all these elements are highly conserved within the Tae4 family (Figure 2E). Moreover, once we mapped this sequence conservation onto the molecular surface of Tae4, we found that these conserved features exclusively cluster around the active site (Figure 2B). The high amino acid conservation and the location of Tae4’s unique structural elements support the notion that this crevice is the key element determining Tae4’s specificity for the acceptor stem side of cross-linked Gram-negative peptidoglycan.

### Periplasmic Peptidoglycan Hydrolase Activity Leads to Inhibition of Bacterial Growth

**Figure 3:**
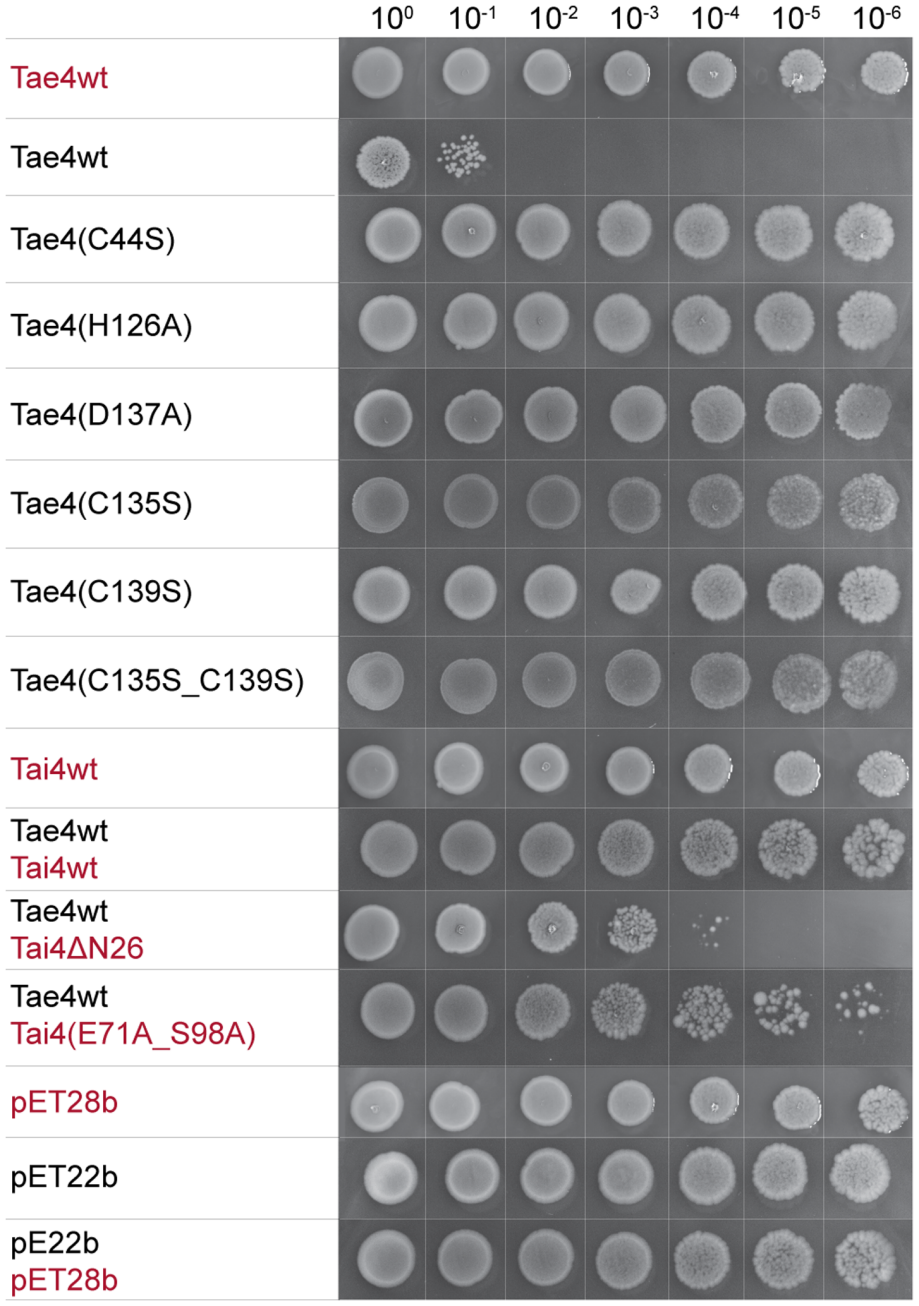
Growth of *Escherichia coli* expressing wild-type and mutated variants of Tae4 and Tai4 proteins. Inoculi were prepared by serial dilutions from 10^0^ to 10^−6^ of overnight cultures and spotted with decreasing optical density from left to the right onto LB-agar plates containing IPTG to induce protein expression. The effector protein Tae4 leads to a significant reduction of bacterial growth upon periplasmic localization. Expression of variants mutated in the active site residues Cys44, H126 and D137 in Tae4 to alanine residues did no more interfere with bacterial growth. A replacement of Cys135 and Cys139 by serine residues did not affect bacterial growth compared to wild-type Tae4. Wild-type Tai4 could rescue the growth defect induced by periplasmic localization of the Tae4. In contrast, a Tai4 variant missing its periplasmic leader sequence (Tai4ΔN26) could no more counteract the growth defect. Additionally, mutations in the effector/immunity interface in Tai4(E71A_S98A) did not rescue the growth phenotype as efficient as the wild-type protein. Proteins which were expressed from pET22b vector constructs contained the pelB leader sequence for artificial periplasmic localization and are labeled in black. Proteins which were expressed from pET28b vector constructs are labeled in red. Vector controls can be found at the bottom of the panel.

Having characterized the Tae4 active site in structural detail we next set out to determine whether the proposed catalytic important residues of Tae4 from *S. typhimurium* evoke a lytic phenotype in *Escherichia coli*. It has already been reported that the Tae4 effector protein as well as the other characterized T6SS effector proteins are only toxic when located in the periplasmic space of Gram-negative bacteria [3], [4]. Therefore, we artificially shuttled Tae4 in the periplasm of *E. coli.* Indeed, whereas cytoplasmic localized *S. typhimurium* Tae4did not interfere with cell proliferation, cell growth was impaired when exporting Tae4 into the periplasm (Figure 3).

In support of a catalytic dyad being the central motif involved in peptidoglycan hydrolysis, variants of Tae4 which had either Cys44 or His126 replaced by an alanine residue did not interfere with bacterial growth anymore (Figure 3). In fact, these two residues are conserved among all reported effector proteins [4] and also have been shown to be essential in Tse1 [14], [16], [20].

Mutation of the third conserved residue Asp137 in *S. typhimurium* Tae4 also abolished the growth phenotype (Figure 3), as observed in *E. cloacae* Tae4 [17]. However, Tse1 contains a cysteine residue at the structurally equivalent position which was shown to have no effect on enzymatic activity [14], [16], [20]. Still, it remains to be shown whether Asp137 is just required for correct positioning of His126 in a catalytic dyad or whether it is involved in a catalytic triad by stabilizing the Nε2-H tautomeric state of His126 similar as reported for serine proteases [26], [27].

A structural superposition of our *S. typhimurium* Tae4 protein structure with the recently reported one [17] showed, that the overall structures are highly similar with the only difference found in the region from residue Cys135 to Phe150. Notably, the electron density for parts of this region was poorly defined in our crystal structure. Two cysteine residues are located in this region, Cys135 and Cys139, which were found to form a disulfide bond in *E. cloacae* Tae4. In contrast, in the crystal structure of Tae4 from *S. typhimurium* no disulfide bond could be observed. These cysteine residues are conserved among homologous Tae4 family members and were shown to be important in Tae4 from *E. cloacae* [17]. Therefore, we wondered whether mutations of these two cysteine residues in Tae4 impair growth of *E. coli* cells after periplasmic localization of Tae4. Indeed, variants with single mutation of either one of this cysteine to a serine residue could no longer evoke the growth phenotype (Figure 3). This effect on the toxicity of Tae4 after mutation of these cysteine residues has also been observed for the *E. cloacae* Tae4/Tai4 system and disulfide bond formation was suggested to be important for correct positioning of the third catalytic aspartate residue [17]. However, although we do not observe any disulfide bond between Cys135 and Cys139 in our crystal structure, the position of Asp137 is virtually identical when compared with the *E. cloacae* homologue. It remains to be shown whether the observed reduced toxicity is indeed due to this disulfide bond as a requirement for enzyme catalysis or whether it serves as protection mechanism against proteolytic degradation.

### A Dimeric Arrangement of Tai4 Immunity Proteins

Whereas the effector molecules are potentially secreted through the injection needle of the type-6-secretion system, immunity proteins are shuttled into the periplasmic region to confer immunity against attacks from siblings and to avoid self-intoxication by self-encoded effector molecules [3]. Thus, we expected Tai4 to be exported and truncated by its periplasmic leader sequence. Indeed, when building the Tai4 polypeptide chain we could model the residues Gln27 to Lys127 in the electron density but could not assign the N-terminal region.

We could verify truncation of the N-terminal leader sequence by mass spectrometry experiments showing that the purified Tai4 had an effective mass of 11,695 Da, which is in excellent agreement with the predicted mass of Tai4 truncated after Ala26 (11,694 Da). Cleavage at this position is most likely, since the upstream region from Gln27 contains a classical periplasmic localization sequence and residues that are essential for efficient export are conserved [28]. In support of a periplasmic localization of Tai4 we showed that only full-length Tai4 could completely rescue the growth phenotype after export of Tae4 in the periplasmic compartment. In contrast, the Tai4ΔN26 variant could only mildly counteract Tae4 toxicity (Figure 3). Notably cells still grow slightly better when Tai4ΔN26 is present in the cytoplasm when compared with cells that express periplasmic Tae4 alone (Figure 3). This partial rescue is most likely due to strayed inhibitor molecules that enter the periplasm upon partial cell lysis by periplasmic Tae4. Thus, our structural model consists of a fully processed Tai4 molecule that had been exported into the periplasm.

**Figure 4:**
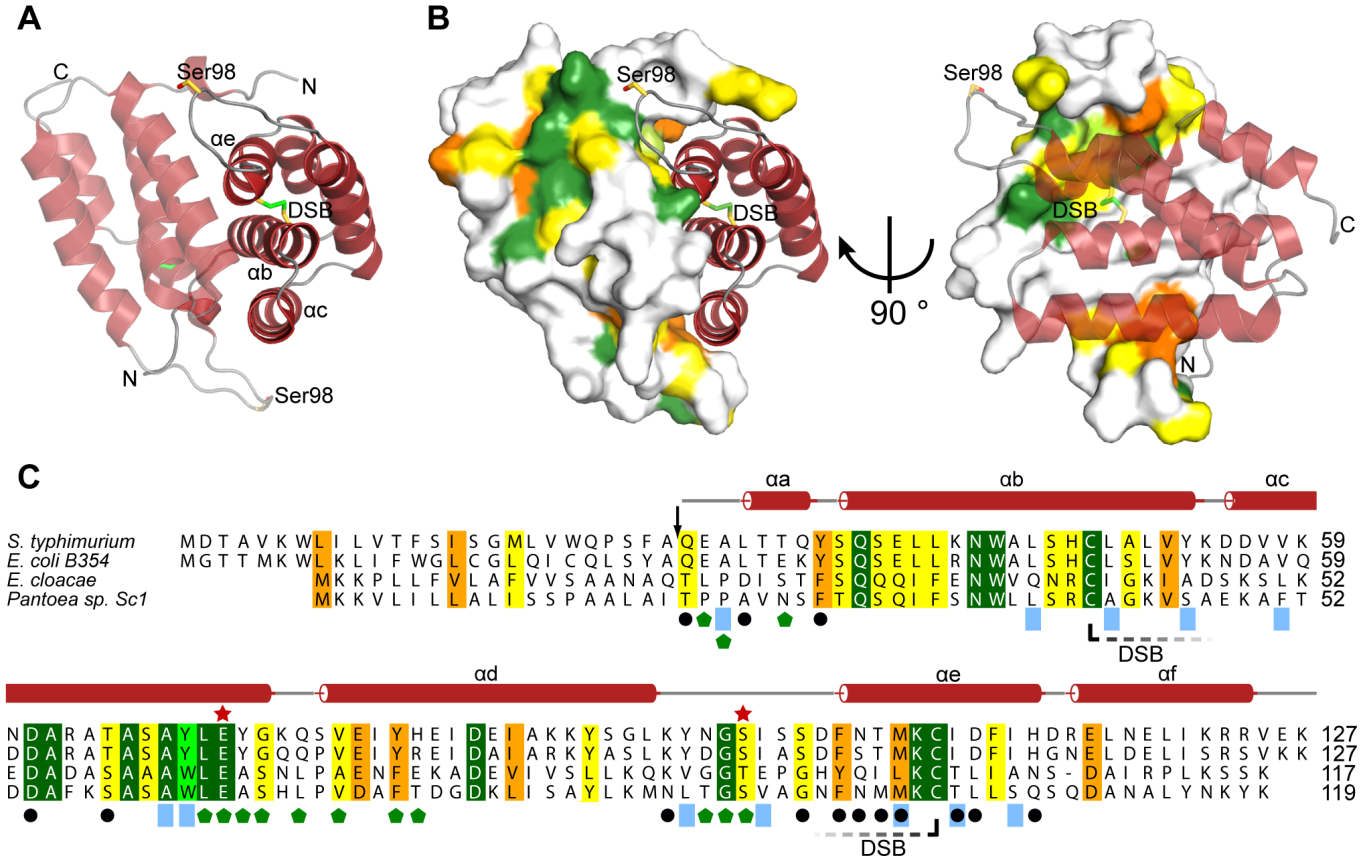
Crystal structure and conservation of the Tai4 immunity protein from *S. typhimurium*. (A) Ribbon representation of Tai4. Tai4 is a purely α-helical protein forming a head-to-tail dimer in the crystal structure. The disulfide bond (DSB) formed by Cys48 and Cys108 that links the α-helices b and e with each other is shown as stick representation. Ser98 which is important for effector inhibition is depicted as stick representation as well. (B) Dimeric arrangement of the Tai4 inhibitor showing the molecular surface of one of the immunity proteins. Amino acid sequence conservation (C) is mapped on the molecular surface. (C) Amino acid sequence alignment of Tai4 immunity proteins from *Salmonella typhimurium* (NP_459276.1), *Escherichia coli* B354 (ZP_06652155.1), *Enterobacter cloacae* (YP_006478115.1) and *Pantoea sp. Sc1* (ZP_09929550.1) going from dark green (identical residues) to light green, orange and yellow (with decreasing conservation). Secondary structure elements above the sequence alignment are colored according to (A). Ser98, located in the inhibition loop between the α-helices d and e as well as the conserved Glu71 residue which makes extensive contacts to Tae4 are marked with a red star. Residues which interact with Tae4 are marked with a green pentagon. Residues that are involved in Tai4 dimer formation by either hydrophobic interactions (blue rectangles) or hydrogen bonds (black dots) are also indicated below the sequence alignment. DSB formation between Cys48 and Cys108 is represented as a dashed line. Cleavage site of the periplasmic leader sequence in Tai4 is indicated with a black arrow above the sequence.

The overall structure of the Tai4 immunity protein showed a purely α-helical fold consisting of six helices stacked onto each other (Figure 4A). Strikingly, a closer inspection of the electron density map revealed that one side formed by the α-helices b, c and e of Tai4 makes extensive contact with the identical surface of a crystal-symmetry related molecule (Figure 4A). The two molecules are in a head-to-tail arrangement and related by a crystallographic twofold symmetry. Each molecule establishes a contact surface of 1410 Å^2^ and the oligomeric state within the biological assembly of Tai4 is therefore most likely dimeric. Whereas the center of this dimer interface is mainly hydrophobic, the boundary area is formed by hydrogen bonds between the two molecules. Additional support for the biological relevance of this dimer interface comes from our bioinformatic studies, showing that the hydrophobic nature of the interface as well as residues that form hydrogen bonds are conserved (Figure 4B and C).

**Figure 5:**
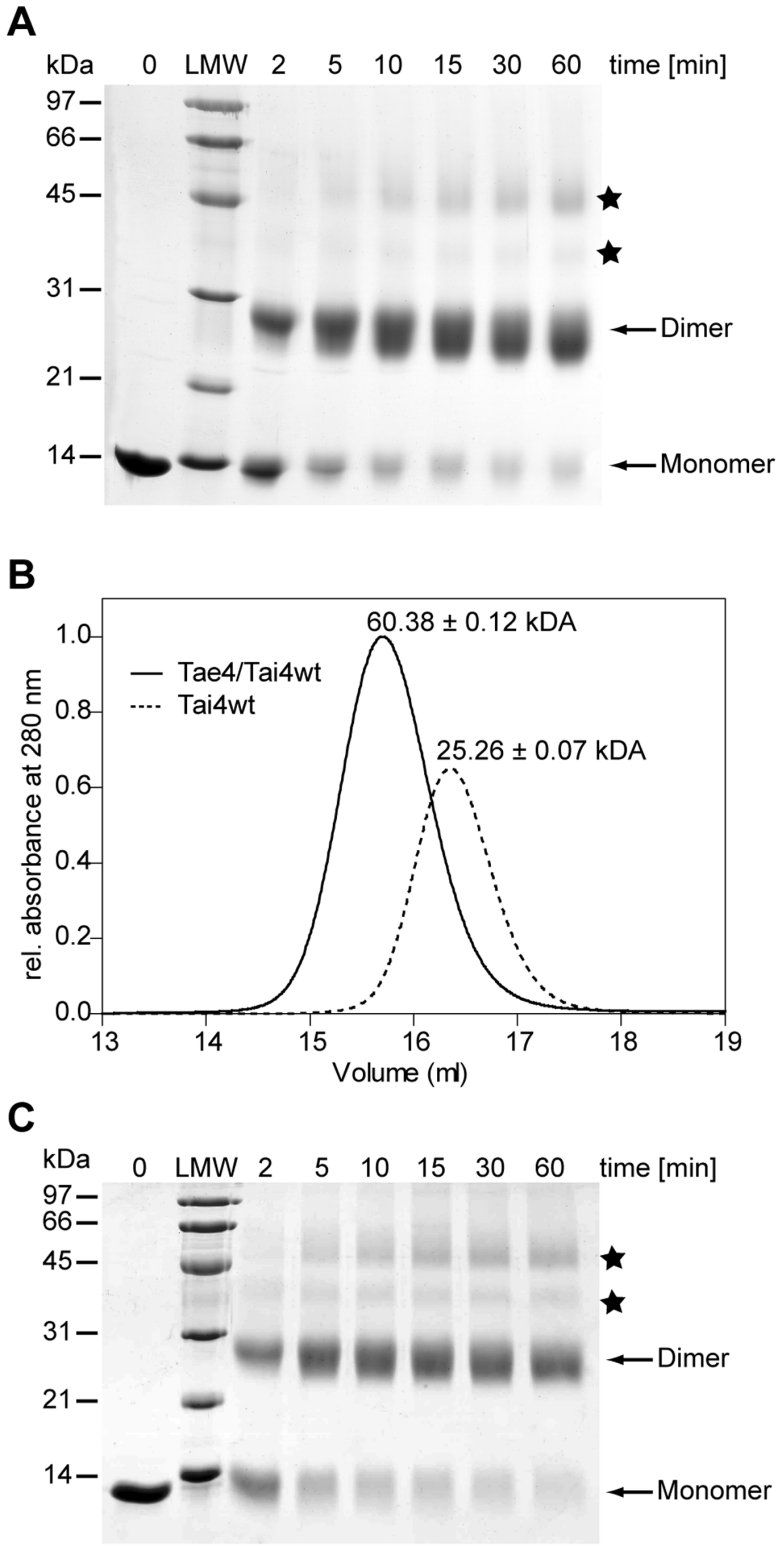
Biological assembly of Tai4 and the Tae4/Tai4 complex. (A) Cross-linking experiments of wild-type Tai4 using glutaraldehyde. Aliquots of cross-linked Tai4 have been taken at different time points (0 to 60 min) and separated on Coomassie stained SDS-PAGE. Bands of monomeric Tai4 with an electrophoretic mobility corresponding to 13 kDa as well as increasing amounts of cross-linked dimeric species of Tai4 (26 kDa) were observed. Note, that minor traces of unspecific intermolecular cross-links are observed (marked with a star) which result in apparent trimeric and tetrameric Tai4 species. LMW: low molecular weight marker in kDa. (B) Gel-filtration and static light scattering of the Tae4/Tai4 effector/immunity complex (solid line) as well as of the wild-type Tai4 immunity protein (dotted line). According to gel-filtration experiments using a gel-filtration standard from BioRad the proteins elute at a corresponding molecular weight of approximately 25 kDa for Tai4 and 46 kDa for the Tae4/Tai4 complex. However, using a multi-angle static light detector averaged molecular masses of 25.26±0.07 kDa for the Tai4 protein and 60.38±0.12 kDa for the Tae4/Tai4 protein complex could be determined. Note that the discrepancy between the masses calculated from the gel-filtration and the static light scattering experiments most likely are caused by the elongated shape of the Tae4/Tai4 complex as observed in the crystal structure. (C) Cross-linking experiments of mutated Tai4(E71A_S98A) using glutaraldehyde have been performed similar to wild-type Tai4 (A).

To further support that Tai4 is predominantly dimeric in solution, we performed glutaraldehyde-cross-linking experiments which showed that indeed purified Tai4 forms dimers (Figure 5A). Ultimately, we showed Tai4 to be dimeric using static light scattering experiments and determined the molecular mass of Tai4 to be 25.26±0.07 kDa. This result is in excellent agreement with the calculated molecular mass for a Tai4 dimer (25.95 kDa) in solution (Figure 5B). Thus, it is rather unlikely that the observed dimer interface is a crystallization artifact.

A DALI search [19] revealed evident homology to the related Tai4 from *E. cloacae* [17] (r.m.s.d of 1.9 Å and 22% sequence identity) and showed homology to Rap1b (r.m.s.d of 1.9 Å and 28% sequence identity) and Rap2b (r.m.s.d of 1.8 Å and 27% sequence identity) from *Serratia marcescens* [11]. Based on the amino acid sequence conservation to the immunity proteins of Rap1a and Rap2a from *S. marcescens* these two proteins have been assigned as immunity proteins as well. Notably, Rap1a together with Ssp1 and Rap2a with Ssp2 form two mutually exclusive effector/immunity pairs of a type-6-secretion system in *S. marcescens* [11]. It is noteworthy that Rap1b as well as Rap2b have been described as dimeric proteins, showing that the dimer formation within this family of immunity proteins is conserved.

A second important feature of Tai4 is a disulfide bond formed by the cysteine residues Cys48 and Cys108. This disulfide bond tethers the N-terminal region of the Tai4 polypeptide chain to the C-terminus by covalently linking α-helix b and e together (Figure 4A). Notably, these two cysteine residues are conserved in all related Tai4 immunity proteins (Figure 4C) and were also observed in Rap1b and Rap2b [11].

### The Tae4_2_/Tai4_2_ Effector/immunity Complex: Inhibition of Tae4

The only available structural information for a different effector/immunity pair of the type-6-secretion system was the crystal structure of Tse1/Tsi1 from *P. aeruginosa* to date [14], [15], [16]. Notably, whereas the effector molecules Tae4 and Tse1 show significant structural similarities, the immunity proteins Tai4 and Tsi1 are unrelated to each other. Here we show that Tai4 from *S. typhimurium* is composed of a stack of α-helices, whereas Tsi1 was reported to be formed by a sandwich of β-sheets [14], [15], [16]. This indicates that immunity proteins of different T6SS families are not derived from a common ancestor. In contrast, the effector molecules do possess a common fold and most likely have evolved from an ancient peptidoglycan hydrolase.

**Figure 6:**
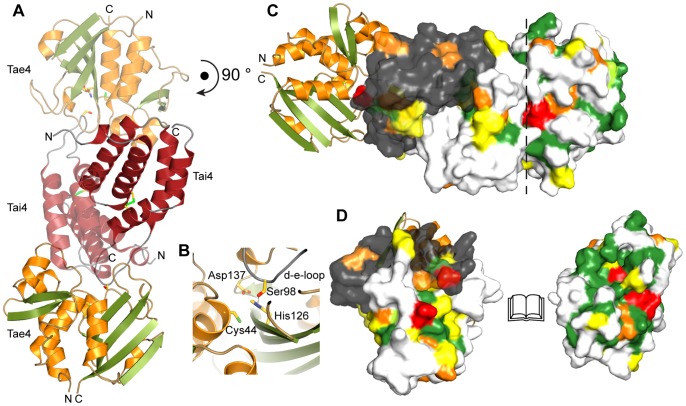
Cyrstal structure of the Tae4/Tai4 heteroteramer. (A) The two symmetry related Tai4 molecules (red transparence and opaque) form a heterotetramer with two Tae4 molecules (orange and green) in our crystal structure. (B) Close up of the interaction between Tae4 and Tai4 at the Tae4 active site. Tai4 inserts the loop region between α-helix d and e (d-e-loop) into the active site of Tae4. This loop harbors Ser98 that forms a hydrogen bond to the catalytic important His126 residue and thereby prevents deprotonation of Cys44. (C) Molecular surface representation of the Tai4 dimer (one monomer colored in white and the second in gray) bound to Tae4 with amino acid sequence conservation mapped onto the molecular surface and colored according to 2E and 4C. Ser98 in Tai4 which has been mutated in this study and its interacting residue in Tae4, His126, are colored in red. The second mutated residue in Tai4, Glu71, and its interacting residues in Tae4, Val80 and Asn81, are highlighted in the red as well. These residues are part of the hydrogen bond network between Tae4 and Tai4. (D) “Open-Book view” of the Tae4/Tai4 heterotetramer interface (as indicated by a dashed line in (C)) with amino acid sequence conservation mapped onto the molecular surface.

Although the immunity proteins are diverse, they apply a common mode of effector active site inhibition. Similar to Tsi1, Tae4 locks up the active site by inserting the loop region between the α-helices d and e (d-e-loop) into the active site and thereby prevents substrate binding and catalysis (Figure 6A). Most importantly, the hydroxyl group of Ser98 in this loop forms a hydrogen bond to the Nδ atom of the catalytic His126 of Tae4, preventing it from deprotonating the catalytic Cys44 residue (Figure 6B). Strikingly, the crystal structure of Tse1/Tsi1 revealed a similar hydrogen bond between a serine residue in Tsi1 in this loop and the catalytically important histidine residue in Tse1 [14], [15], [16]. Moreover, this hydroxyl group is conserved in all Tai4 members (Figure 4C) and was also found in the crystal structure of Tae4/Tai4 from *E. cloacae* [17]. Apparently, immunity proteins have evolved by convergent evolution to prevent formation of a reactive thiolate ion at the active site cysteine in the effector/immunity protein complex.

Apart from blocking the active site and preventing thiol formation, the overall mode of inhibition differs between Tai4 and Tsi1. In contrast to the Tse1/Tsi1 complex which has been reported to be a heterodimer [14], [16], we found that the inhibited Tae4/Tai4 protein complex from *S. typhimurium* forms heterotetramers. In this heterotetrameric assembly, the two polypeptide chains of a Tai4 homodimer inhibit two Tae4 molecules. In more detail, the d-e-loop of one Tai4 polypeptide chain together with α-helix c and the N-terminal region of α-helix d of the second Tai4 molecule in the homodimer establish a contact surface of 1144 Å^2^ with each individual Tae4 effector molecule (Figure 6C and D). α-Helix c in particular plays a pivotal role in inhibition, since it holds the d-e-loop that occludes the active site in place but also closes over the putative muropeptide binding site and prevents binding. Most importantly, the majority of the residues that form this effector-immunity protein interaction site are conserved within Tai4 proteins (Figure 4C) and a similar mode of inhibition has also been described for the Tae4/Tai4 effector immunity system from *E. cloacae* [17]. This indicates that Tai4 in its dimeric form has evolved to exclusively inhibit Tae4 related proteins. In particular, potentially monomeric Tai4-related immunity proteins will not sufficiently inhibit Tae4. Dissection of the interaction surface to the individual contribution of the two polypeptide chains revealed that they are most likely too small and thus the interaction too weak for inhibition (362 Å^2^ are contributed by the loop region inhibiting the active site and only 810 Å^2^ are contributed by the second polypeptide chain of Tai4).

We could further corroborate this heterotetrameric arrangement of the Tae4/Tai4 protein complex by static light scattering experiments, which showed that the purified complex has a molecular mass of 60.38±0.12 kDa (Figure 5B). This molecular mass is in good agreement with the calculated molecular mass for a Tae4_2_/Tai4_2_ heteroteramer (62.07 kDa). Finally, we could also demonstrate the importance of heterotetramer formation *in vivo*. Whereas the growth phenotype of periplasmic localization of Tae4 in *E. coli* could be rescued by co-expression of wild-type Tai4, a mutated Tai4 variant (E71A_S98A) could no longer completely complement the growth defect (Figure 3). These mutations are in the d-e-loop and in α-helix c and are thus located on opposing sites at the surface of a Tai4 monomer. Therefore, the two residues are contributed from two different polypeptide chains within an individual Tae4/Tai4 interface. We can further exclude that these mutations interfere with dimer formation of Tai4, since they can still be effectively cross-linked with each other (Figure 5C). Thus, the failure in fully rescuing the growth defect after periplasmic localization of Tae4 is most likely due to a weaker affinity of a Tai4 homodimer for Tae4. In fact, Ser98 of Tai4 forms a hydrogen bond to the catalytic important His126 residue in Tae4 and Glu71 in Tai4 makes extensive contacts to residue Val80 and Asn81 in Tae4 by hydrogen bonds (Figure 6D).

**Figure 7:**
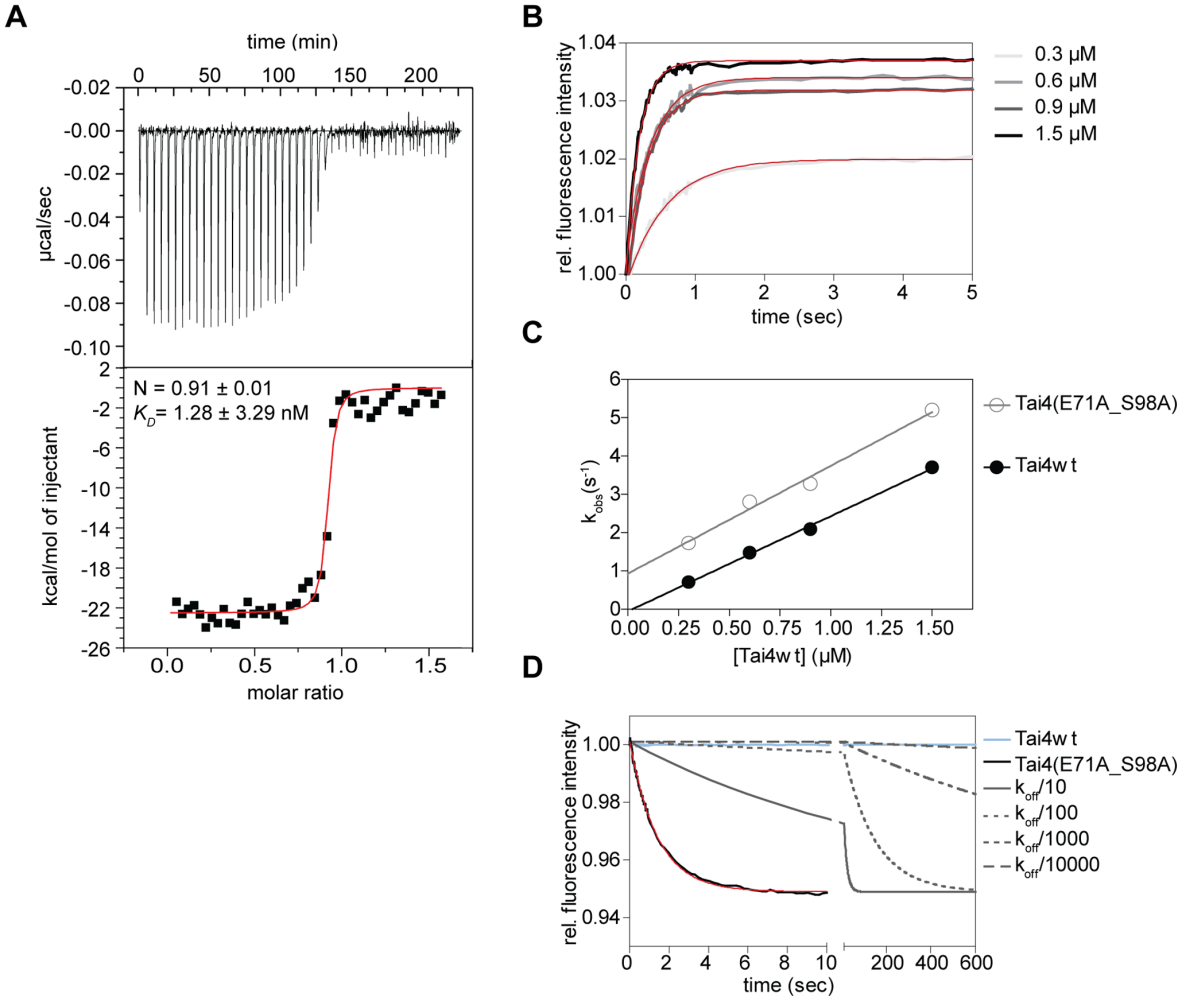
Affinity and association/dissociation kinetics of the Tae4/Tai4 complex. (A) Isothermal titration calorimetry experiment. The binding constant (*K*_D_), as well as the stoichiometric ratio (N) are determined by fitting a single binding-site model to the data. Original titrations are shown above the fit. (B) Kinetic traces of Tae4-ATTO 488 and wild-type as well as mutated Tai4(E71A_S98A) binding have been recorded at different Tai4 concentration (0.3–1.5 µM from light gray to black). The time has been plotted against the relative fluorescence signal and single exponential functions have been fitted to the data (red). The time traces and the fits are exemplarily shown for binding of the Tai4(E71A_S98A) protein (C) The observed rate constants k_obs_ from (B) have been plotted against the concentrations of Tai4wt (filled black circles) and Tai4(E71A_S98A) (empty gray circles) and fitted using a linear regression (Tai4wt:black, Tai4(E71A_S98A):gray). The association rate constants of Tai4 binding could be extracted from the slopes of the linear functions. Although the slope of the linear functions is just slightly increased for Tai4(E71A_S98A), the y-axis intercept (corresponding to the off-rate) is significantly lower for wild-type Tai4. (D) The dissociation rate constants were determined separately by dissociation experiments using unlabelled Tae4 protein to chase off the Tae4-ATTO 488/Tai4 complex. Shown are the time traces recorded from 1 µM Tae4 to chase off the effector/immunity complex. The time trace for wild-type Tae4/Tai4 complex is shown in blue. The traces for the mutated Tae4/Tai4 protein complex is shown in black with the corresponding exponential fit in red. Simulated time traces using the exponential fit values of the Tai4(E71A_S98A) experiment with the dissociation rate constant divided by up to a factor of 10^4^ are depicted in gray. Simulated time traces suggested an off-rate between 0.7 ×10^−3^ and 0.7 ×10^−4^ s^−1 ^for wild-type Tai4; although, it can not be excluded to be lower. All values obtained from the stopped-flow experiments are shown in Table 2.

In order to characterize the affinity of Tae4 for Tai4 in the effector immunity protein complex we performed isothermal titration calorimetry (ITC) experiments. However, even when we reduced the protein concentrations to the lowest possible at which a sufficient signal to noise ratio for individual titrations could be detected, we still did not obtain data that enabled an exact determination of the binding constant for the protein complex (Figure 7A). Thus we could only conclude from these experiments, that the *K_D_* had do be lower than 1 nM. Therefore, we moved on and performed stopped-flow experiments measuring the transient kinetics of Tae4/Tai4 binding (Figure 7). Strikingly, when we mixed fluorescently labeled Tae4 with wild-type or the Tai4(E71A_S98A) variant, similar time traces were observed (Figure 7B). In fact, the association rate constant of 2.485±0.081 µM^−1^s^−1^ determined for wild-type Tai4 protein is in a similar range as the association rate constant obtained for the Tai4(E71A_S98A) variant with 2.812±0.216 µM^−1^s^−1^ (Figure 7B and C, Table 2). Apparently, the association rate constant of the Tai4 protein to the effector protein is not impaired by the E71A and S98A mutations. In order to exclude that the measured association rate constants are significantly perturbed by the fluorescence label, rapid mixing kinetics were recorded monitoring the change in intrinsic tryptophan fluorescence upon Tae4/Tai4 association. Indeed, the determined association rate constant of 2.566±0.233 µM^−1^s^−1^ is similar to what was observed for labeled Tae4 protein (data not shown).

**Table 2:** Affinity constants for wild-type and mutant Tai4 protein binding to Tae4.

Tai4 variant	Accociation rate k_on_ (µM^−1^s^−1^)	Dissociation rate k_off_ (s^−1^)	Binding constant *K_D_* (µM)
Tai4wt	2.485±0.081	<0.7×10^−3^.	<0.28×10^−3^
Tai4_E71A_S98A	2.812±0.216	0.716±0.003	0.255±0.014

When we chased off fluorescently labeled Tae4 from a preassembled Tae4/Tai4(E71A_S98A) complex, we were able to determine an apparent dissociation rate constant of 0.716±0.003 s^−1^ (Figure 7D and Table 2). We therefore could determine the binding constant of the Tae4/Tai4(E71A and S98A) complex to be 0.255±0.014 µM calculated by the ratio between k_off_ and k_on_ (Table 2). In contrast, when we tried to chase off Tae4 in complex with wild-type Tai4, no change in the fluorescence signal could be observed even after 12 h of incubation. However, using the fitting parameters obtained from the Tai4(E71A_S98A) displacement data for off-rate simulations of the wild-type Tai4 protein we can predict, that the off-rate for the wild-type Tae4/Tai4 complex must be at least three to four orders of magnitude lower (Figure 7D) and so must be in the sub-nanomolar regime. This result is comparable with the reported binding constants for Tae4/Tai4 from *E. cloacae* [17]. Notably, the reported binding constant for the Tai4(E71A_S98A) double mutant is significantly higher than the reported values for the single mutations for the *E. cloacae* system. This clearly demonstrates that the effect of the double mutation is synergistic and thus both residues contribute to binding. Furthermore, our structural results show that within one Tae4/Tai4 interface of a heterotetrameric assembly these residues originate from the two polypeptide chains of a Tai4 homodimer with the Tae4/Tai4 complex.

### Conclusion

Our work provides insights into the different cleavage specificities of the *S. typhimurium* Tae4 effector compared to the Tse1 effector from *P. aeruginosa*. Tae4 and Tse1 are structurally related in their NlpC/P60 core but differ in structural elements adjacent to their catalytic centers. Moreover, residues of these Tae4 specific elements cluster at a patch on the molecular surface of Tae4 that is highly conserved within the family of Tae4 effectors. Thus, these highly conserved elements are most likely important for the specificity of the acceptor stem of cross-linked muropeptides. However, in order to unambiguously identify the molecular mechanisms responsible for substrate specificity, structural information on the effector proteins bound to their substrates is required.Tae4 and Tai4 form a heterotetramer which seems to be conserved among homologous effector/immunity proteins. Taken together, Tae4 and Tai4 is a new type of effector/immunity proteins and our studies further deepen the current knowledge of T6SS substrates which could be used to develop new antimicrobial therapies.

## Materials and Methods

### Cloning

A bicistronic expression construct harboring a *S. typhimurium* full-length *Tae4 orf* (NP_459275.1) followed by the *Tai4 orf* (NP_459276.1) was synthetically manufactured by GeneArt® (Life Technologies). The *Tae4 orf* was engineered to be encoded downstream from an NcoI restriction site fused with a hexahistidine-encoding DNA sequence. An MfeI restriction site was included downstream from the stop codon of *Tae4* for further subcloning steps. The *Tai4 orf* was placed downstream from an individual ribosomal binding site and fused with an NdeI restriction site in frame with its start codon. Downstream from the stop codon of *Tai4* an EcoRI restriction site was included. Subsequently to synthesis, the bicistronic operon was re-cloned into a pET28b vector (Novagen) using the NcoI and EcoRI restriction sites. For single expression constructs, DNA of the gene encoding full-length Tae4 was obtained by digestion of DNA of the bicistroinc expression construct using the 5′-NcoI and 3′-MfeI restriction enzymes and subsequently ligated into a pET28b vector. An expression construct encoding for full-length Tai4 fused with a C-terminal hexahistidine tag was obtained by PCR amplification from the bicistronic expression construct using the primers tai4_NcoI_f and tai4_NotI_r (Table S1) that subsequently was cloned into a pET28b vector. The expression construct for the Tai4ΔN26 variant that lacks the periplasmic leader sequence was created by PCR amplification using the primers tai4ΔN26_NcoI_f and tai4_NotI_r (Table S1) followed by cloning into a pET28b vector. For artificial periplasmic localization of Tae4, DNA of the Tae4 gene was amplified by PCR using the primers tae4_NcoI_f and tae4_NotI_r (Table S1) and ligated into a pET22b vector DNA (Novagen) resulting in a fusion-protein that contains an N-terminal pelB leader sequence. Mutants of Tae4 and Tai4 were prepared using the QuikChange protocol from Stratagene using the primers listed in Table S1.

### Protein Expression and Purification

*Escherichia coli* BL21-CodonPlus(DE3)-RIL cells (Stratagene) were either transformed with the bicistronic or the individual Tae4 or Tai4 expression constructs. For protein expression the transformed *E. coli* cells were grown at 310 K in LB-medium containing kanamycin (50 µg/ml) and chloramphenicol (34 µg/ml). After cells were grown to mid-log phase (OD_600_ ∼0.6) the temperature was reduced to 293 K and protein expression was induced by addition of 0.5 mM isopropyl-β-*D*-1-thiogalactopyranoside. Cells were harvested 18 h after induction and resuspended in their individual, ice cold resuspension buffer (see below). All subsequent steps were performed at 277 K. Cells were lysed by sonication and the supernatant clarified by centrifugation.

For purification of the Tae4/Tai4 complex, the cell pellets were resuspended in buffer A (50 mM Tris-HCl pH 7.5, 200 mM NaCl, 50 mM (NH_4_)_2_SO_4_, 3 mM 2-mercaptoethanol), cells were lysed and the clarified lysate was incubated with 1.5 ml talon metal affinity resin (Clonetech) equilibrated with buffer A. After 3 h of incubation, the resin was washed with buffer A and bound proteins were eluted with buffer B (50 mM Tris-HCl pH 7.5, 200 mM NaCl, 50 mM (NH_4_)_2_SO_4_, 150 mM imidazole, 3 mM 2-mercaptoethanol). Subsequently, the protein was diluted 1∶3 with buffer C (50 mM Tris-HCl pH 7.5, 200 mM NaCl, 50 mM (NH_4_)_2_SO_4_, 2 mM dithioerythritol (DTE)) and dialyzed overnight against buffer C. The dialyzed protein was further diluted 1∶3 with buffer D (50 mM Tris-HCl pH 8.0, 50 mM NaCl, 2 mM DTE) and loaded onto a MonoQ 10/100 GL column (GE Healthcare) equilibrated with buffer D. The flow-through, which contained the Tae4/Tai4 complex was collected and concentrated by ultrafiltration. Subsequently, the complex was applied to a Superdex75 10/300 GL column (GE Healthcare) equilibrated with buffer E (50 mM HEPES-NaOH pH 8.0, 200 mM NaCl, 1 mM *tris*(2-carboxyethyl)phosphine). The eluted fractions were again concentrated by ultrafiltration to 16 mg/ml, as determined by measuring the absorbance at 280 nm (ε_280_ = 59,360 cm^−1^ M^−1^). The concentrated protein was either flash-cooled in liquid nitrogen and stored at −80°C or subsequently used for crystallization experiments. Selenomethionine-subsituted Tae4/Tai4 complex was expressed according to Van Dyne *et al.* [29]. The labeled Tae4/Tai4 protein complex was purified essentially as described for the native protein complex except that immediately after elution from the metal affinity column the protein complex was diluted 1∶10 with buffer D before loading onto the MonoQ column. Furthermore, all buffers used for purification of the substituted protein complex contained additional reducing agent (buffer A/B: 5 mM 2-mercaptoethanol, buffer D: 4 mM DTE, buffer E: 3 mM *tris*(2-carboxyethyl)phosphine). The selenomethione-substituted Tae4/Tai4 protein complex was concentrated to 12 mg/ml and used for crystallization.

For purification of full-length Tae4, the cell pellets were resuspended in buffer F (50 mM MES pH 6.5, 200 mM NaCl, 50 mM (NH_4_)_2_SO_4_, 3 mM 2-mercaptoethanol), cells were lysed and the clarified lysate was incubated with 2.0 ml talon metal affinity resin. After 3 h of incubation, the resin was washed with buffer F followed by a high salt wash with buffer G (50 mM MES pH 6.5, 1 M NaCl, 50 mM (NH_4_)_2_SO_4_, 3 mM 2-mercaptoethanol) and bound proteins were eluted with buffer H (50 mM MES pH 6.5, 200 mM NaCl, 50 mM (NH_4_)_2_SO_4_, 150 mM imidazole, 3 mM 2-mercaptoethanol). Subsequently, the protein was diluted 1∶5 with buffer I (50 mM MES pH 6.5, 10 mM (NH_4_)_2_SO_4_, 2 mM DTE) and loaded onto a MonoS 5/50 GL column (GE Healthcare). The protein was eluted in a linear gradient of 15 column volumes to buffer J (50 mM MES pH 6.5, 1 M NaCl, 10 mM (NH_4_)_2_SO_4_, 2 mM DTE). The concentrated protein was applied to a Superdex75 10/300 GL column (GE Healthcare) equilibrated with buffer K (50 mM MES pH 6.5, 200 mM NaCl). The eluted fractions were again concentrated by ultrafiltration to 4 mg/ml, as determined by measuring the absorbance at 280 nm (ε_280_ = 32430 m^−1^ M^−1^). The concentrated protein was either flash-cooled in liquid nitrogen and stored at -80°C or subsequently used for experiments.

For purification of periplasmic Tai4, cell pellets were resuspended in buffer A, cells were lysed and the clarified lysate was incubated for 3 h with 2 ml talon metal affinity resin. After incubation the resin was washed with buffer A and bound proteins were eluted with buffer L (50 mM Tris-HCl pH 8.0, 150 mM imidazole, 3 mM 2-mercaptoethanol). Subsequently, the protein was 1∶5 diluted with buffer M (50 mM Tris-HCl pH 8.0, 2 mM DTE) and loaded onto a MonoQ 10/100 GL column. The protein was eluted in a linear gradient of 10 column volumes to buffer N (50 mM Tris-HCl pH 8.0, 1 M NaCl, 2 mM DTE) and concentrated by ultrafiltration. The concentrated protein was applied to a Superdex75 10/300 GL column (GE Healthcare) equilibrated with buffer K. The eluted fractions were again concentrated by ultrafiltration to 19 mg/ml, as determined by measuring the absorbance at 280 nm (ε_280_ = 15930 m^−1^ M^−1^). The concentrated protein was either flash-cooled in liquid nitrogen and stored at -80°C or subsequently used for experiments. The mutated Tai4_E71A_S98A variant protein was purified using the identical protocol (final concentration 28.5 mg/ml). Protein purity during all purification procedures was monitored by Coomassie-stained SDS-PAGE and the final protein batches were judged to be 99% pure.

### Gel-filtration Analysis Coupled to Static Light Scattering Experiments

Gel-filtration experiments were performed on a Waters HPLC system (Milford) using a tandem separation/detection setup of a Superdex200 10/300 GL column (GE Healthcare) connected to a refractive index detector 2414, a photodiode array detector 2996 and a multiangle light scattering detector (Dawn Heleos, Wyatt). The system was equilibrated with buffer O (50 mM Tris-HCl pH 8.0, 200 mM NaCl). The purified Tae4/Tai4 protein complex and Tai4 protein was diluted in buffer O to a final concentration of 100 µM. 40 µl of the protein solution was applied to the system at a flow rate of 0.5 ml/min. The setup was calibrated using a well-defined gel-filtration standard (Bio-Rad, Order no.151–1901) and thus, determination of exact molecular masses was possible. Data analysis of the light scattering data was performed using the ASTRA software provided by the manufacturer (Wyatt).

### Tai4 Cross-linking Experiments

Cross-linking of Tai4 wild-type as well as the mutated protein variant was performed using glutaraldehyde. Briefly, 10 µl of a 2.3% (*v/v*) glutaraldehyde solution was added to 200 µl protein solution at a concentration of 0.25 µg/µl in 20 mM HEPES pH 7.5 buffer. The cross-link reactions were incubated at 37°C, 20 µl aliquots were taken at different time points and quenched by adding 2 µl of 1 M Tris-HCl pH 8.0. Finally, cross-linking efficiency was analyzed by Coomassie-stained SDS-PAGE.

### Fluorescence Labeling of Tae4

Purified effector protein Tae4 was labeled with an ATTO 488 fluorescence label (ATTO-TEC GmbH) according to the manufacture’s instruction. Briefly, 1 ml of Tae4 effector protein at a concentration of 1.5 mg/ml in buffer K was supplemented with thiol-reactive ATTO 488 maleimid dye at equimolar concentration. The labeling reaction was incubated for 2 h at room temperature and quenched by adding 500 µl buffer K supplemented with 2 mM DTE. Unbound dye was removed with a NAP25 desalting column (GE Healthcare) equilibrated with buffer K supplemented with 2 mM DTE. After elution with the equilibration buffer, the labeled protein was dialyzed overnight against the same buffer. The labeled protein was concentrated and protein concentration was determined by UV/VIS spectroscopy. The labeling efficiency was determined by the ratio of absorbance at 504 nm and 280 nm (corrected by the absorbance of the dye) to be 92%.

### Stopped-flow Experiments

Transient-kinetic measurements of Tae4-ATTO 488 and wild-type as well as mutated Tai4 protein variants were performed at 293 K in buffer K using a BioLogic SFM-400 stopped-flow instrument (BioLogic Science Instruments). The excitation wavelength was set to 485 nm and the emission of the fluorescence signal was measured using a 515 nm long path filter (LP515FG05-25, LOT Oriel group). Direct mixing kinetics to determine the on-rate were performed with 0.3 µM Tae4-ATTO 488 and 3 µM wild-type as well as mutant Tai4 stock solutions. Time traces were collected at different ratios of 0.1 µM Tae4-ATTO 488 and 0.3–1.5 µM wild-type or mutated Tai4 proteins in the detection cell after mixing. Kinetic data were recorded as triplicates and averaged. Analysis of the data was performed by exponential fitting using GraphPadPrism 5. The observed rate constants were plotted against the wild-type or mutant Tai4 concentration and fitted using a linear regression. The association rate constants of binding were extracted from the slope of the linear function.

To determine the association rate constant using the change in intrinsic tryptophan fluorescence of unlabeled Tae4 and wild-type Tai4 protein upon binding, stopped-flow experiments using an excitation wavelength of 280 nm were performed. The fluorescence signal was measured using a 320 nm long path filter (LP320FG01-25, LOT Oriel group). Tae4 and wild-type Tai4 stock solutions of 3.5 µM and 24 µM, respectively were prepared in buffer K. Time traces were collected at different ratios of 1.8 µM Tae4 and 2.3–9.9 µM wild-type Tai4 in the detection cell after mixing. Analysis of the data was similar as already described for the measurements with labeled Tae4. For dissociation experiments 0.6 µM ATTO 488 labeled Tae4 was pre-incubated with 0.6 µM of the wild-type or mutated Tai4. Labeled Tae4 was chased off from the protein complex using a 4 µM unlabeled protein stock solution. Time traces for chase off experiments were collected at cell concentration ratios of 0.1 µM wild-type or 0.2 µM mutated Tae4/Tai4 complex and 1 µM or 2 µM unlabeled Tae4. The recorded kinetic traces were fitted to single exponential functions and the extracted rate constants correspond to the dissociation rate constant of Tae4 binding.

### Isothermal Titration Calorimetry (ITC)

Isothermal calorimetric titration experiments were performed at 293 K using a VP-ITC MicroCalaorimeter (MicroCal, LLC, Northampton). Stock solutions of Tae4 and wild-type Tai4 were prepared in buffer K and degassed for 5 min at 291 K. The cell was filled with Tae4 protein at a concentration of 2.17 µM into which wild-type Tai4 protein at a concentration of 29.5 µM was titrated. For 45 titrations of 3.5 µl of Tai4 into Tae4 the heat of binding was recorded. Data were extracted and fitted using a single-site binding model following the manufacturer’s instructions.

### Cell Toxicity Analysis

To determine cell toxicity of the effector/immunity proteins *Escherichia coli* BL21(DE3)pLysS cells (Stratagene) were transformed with the pET22b-Tae4 and pET28b-Tai4 wild-type and mutant constructs. A single colony was grown in 10 ml LB media supplemented with the appropriate antibiotics (ampicillin 100 µg/µl, kanamycin 50 µg/l, chloramphenicol 34 µg/µl) at 310 K overnight. Subsequently, these overnight cultures were diluted in LB media to an final OD_600_ of 1.5. Cultures were further diluted in LB media in 10-fold steps to a 10^−6^ dilution. From these dilution series 3 µl aliquots were spotted onto LB-agar plates supplemented with 0.05 mM IPTG and the appropriate antibiotics. Plates were incubated at 310 K for 20 h.

### Mass Spectrometry

The analysis was performed in the linear, positive ion mode with blanking (<600 *m*/*z*) using an Axima Performance MALDI TOF/TOF mass spectrometer (Shimadzu Biotech Germany). All reagents and protein standards were purchased from Sigma-Aldrich. Trans-3,5-dimethoxy-4-hydroxy cinnamic acid (10 mg/ml in 50% acetonitrile, 50% of 0.1% trifluoroacetic acid (all % *v/v*)) was used as a matrix. Sample positions on the steel 384-position sample plate were washed once with matrix solution. A 1-µl drop of the protein preparation (1 mg/ml) was dried on the sample plate at room temperature and washed with a small drop of 0.1% trifluoroacetic acid applied for 10 s. Immediately after removal of the trifluoroacetic acid, 1 µl of matrix solution was added to the sample and it was allowed to dry at room temperature. The standard proteins (about 1 pmol of ubiquitin, cytochrome C, and apomyoglobin) were spotted onto the washed plate and an equal volume (usually 1 µl) of matrix was immediately added to the protein drop. Each protein standard was analyzed separately and a combined calibration of the near external standards was employed to determine the mass/charge (*m*/*z*) values.

### Crystallization and Cryo-protection

Original hits for crystallization conditions were identified by sparse matrix screening in a 96 well format using the JCSG Core Suite (Qiagen, Hilden). Crystallization conditions were further refined in Linbro plates at 293 K in a hanging-drop vapor diffusion setup. Crystals of maximal size were obtained after 3 days when 700 µl of a reservoir solution containing 30% (*v/v*) 2-ethoxyethanol and 200 mM *tri*-sodium citrate at pH 6.0 were applied and a protein to reservoir ratio of 1∶1 was used. Native and selenomethionine-labeled proteins crystallized under identical conditions. Crystals were harvested and washed in reservoir solution which was sufficient as cryo-protectant for flash-cooling in liquid nitrogen.

### Data Collection and Structure Determination

Diffraction data of native as well as selenomethionine-subsituted Tae4/Tai4 crystals were collected at the X10SA beam line of the Swiss Light Source (Villigen) at 100 K. Collected diffraction data were processed using XDS [30]. For both data sets, 5% of the reflections were initially randomly assigned and kept constant for all other datasets. These reflections were excluded during refinement and used to calculate an R_free_ value. From a selenomethionine-labeled Tae4/Tai4 protein crystal a highly redundant SAD data set was collected and used to determine the heavy-atom substructure with SHELXD [31] resulting in an interpretable electron density map into which a model was built manually using COOT [32]. The preliminary model was built and refined in alternating cycles of REFMAC [33] with TLS [34] refinement and manual re-building. The model phases were extended using a rigid body refinement protocol [35] to a resolution of 1.8 Å using the native data and refined similar as described for the selenomethionine dataset. Model quality was evaluated using MolProbity [36] and figures were prepared using PYMOL [37]. Sequence conservation of Tae4 as well as Tai4 homologous proteins was determined using the AMAS-server [38] and illustrated using ALSCRIPT [39]. Molecular interaction surfaces were calculated and analyzed using the PISA server [40] and CONTACT [35]. The atomic coordinates and structure factor amplitudes for both models have been deposited in the Protein Data Bank (PDB) under the accession codes 4J30 (Tae4/Tai4 SeMet) and 4J32 (Tae4/Tai4 native).

## Supporting Information

Table S1 Primer for the Tae4 and Tai4 construct design and site directed mutagenesis of Tae4 and Tai4.
(DOC)
