# Supplementary material for: Structural Insights into the Effector – Immunity System Tae4/Tai4 from Salmonella typhimurium
Source: PLoS One. 2013 Jun 27;8(6):e67362. doi: 10.1371/journal.pone.0067362 (PMC3695027; doi:10.1371/journal.pone.0067362)
Supplement: Table S1 — Primer for the Tae4 and Tai4 construct design and site directed mutagenesis of Tae4 and Tai4. (DOC) [file pone.0067362.s001.doc]

**Supplemental Table S1: Primer for the Tae4 and Tai4 construct design and site directed mutagenesis of Tae4 and Tai4.**

| **Primer** | **Sequence 5’-3’** |
| --- | --- |
| tae4_NcoI_f | GCATACCATGGGAAACAGACCTTCATTCAATG |
| tae4_NotI_r | GCTATGCGGCCGCCGGCAGTATCCACAGTG |
| tae4_C44S_f | CTGGTGGTTATTTTCAAAATGCCTCCCCTATTCGAATGAG |
| tae4_C44S_r | CTCATTCGAATAGGGGAGGCATTTTGAAAATAACCACCAG |
| tae4_H126A_f | CTGGAGCAATGCCAGAGGAGCCGTTACATTATGGAATG |
| tae4_H126A_r | CATTCCATAATGTAACGGCTCCTCTGGCATTGCTCCAG |
| tae4_C135S_f | GGAATGGCAGTATCTCTTCAGATCAGTGCCAC |
| tae4_C135S_r | GTGGCACTGATCTGAAGAGATACTGCCATTCC |
| tae4_D137A_f | GAATGGCAGTATCTGTTCAGCTCAGTGCCACTTATTAAATG |
| tae4_D137A_r | CATTTAATAAGTGGCACTGAGCTGAACAGATACTGCCATTC |
| tae4_C139S_f | GAATGGCAGTATCTGTTCAGATCAGTCCCACTTATTAAATG |
| tae4_C139S_f | CATTTAATAAGTGGGACTGATCTGAACAGATACTGCCATTC |
| tae4_C135SC139S_f | GGAATGGCAGTATCTCTTCAGATCAGTCCCACTTATTAAATGACC |
| tae4_C135SC139S_r | GGTCATTTAATAAGTGGGACTGATCTGAAGAGATACTGCCATTCC |
| tai4_NcoI_f | GCATACCATGGATACTGCCGTGAAATGG |
| tai4dN26_ NcoI_f | GCATACCATGGGACAAGAAGCATTGACCACAC |
| tai4_ NotI_r | CGATAGCGGCCGCCTTCTCGACACGCC |
| tai4_E71A_f | CGGCCAGTGCTTACCTTGCGTATGGTAAACAATCTGTGG |
| tai4_E71A_r | CCACAGATTGTTTACCATACGCAAGGTAAGCACTGGCCG |
| tai4_S98A_f | CAGGGTTGAAATATAACGGTGCGATATCATCAGATTTTAATACC |
| tai4_S98A_f | GGTATTAAAATCTGATGATATCGCACCGTTATATTTCAACCCTG |
